# Predictive value of body mass index to metabolic syndrome risk factors in Syrian adolescents

**DOI:** 10.1186/s13256-017-1315-2

**Published:** 2017-06-25

**Authors:** Mahfouz Al-Bachir, Mohamad Adel Bakir

**Affiliations:** 1 Department of Radiation Technology, Atomic Energy Commission of Syria, P.O. Box 6091, Damascus, Syrian Arab Republic; 2 Department of Nuclear Medicine, Atomic Energy Commission of Syria, P.O. Box 6091, Damascus, Syrian Arab Republic

**Keywords:** Syrian adolescents, Body mass index (BMI), Blood pressure, Metabolic syndrome, Risk factors

## Abstract

**Background:**

Obesity has become a serious epidemic health problem in both developing and developed countries. There is much evidence that obesity among adolescents contributed significantly to the development of type 2 diabetes and coronary heart disease in adulthood. Very limited information exists on the prevalence of overweight, obesity, and associated metabolic risk factors among Syrian adolescents. Therefore, the purpose of this study was to determine the relationship between obesity determined by body mass index and the major metabolic risk factors among Syrian adolescents.

**Methods:**

A cross-sectional study of a randomly selected sample of 2064 apparently healthy Syrian adolescents aged 18 to 19 years from Damascus city, in Syria, was performed. Body mass index and blood pressure were measured. Serum concentrations of glucose, triglycerides, total cholesterol, high-density lipoprotein-cholesterol, and low-density lipoprotein-cholesterol were determined. Metabolic syndrome was defined using the national criteria for each determined metabolic risk factor. Individuals with a body mass index 25 to 29.9 were classified as overweight, whereas individuals with a body mass index ≥30 were classified as obese.

A receiver operating characteristics curve was drawn to determine appropriate cut-off points of the body mass index for defining overweight and obesity, and to indicate the performance of body mass index as a predictor of risk factors.

**Results:**

The obtained data showed that blood pressure and the overall mean concentrations of fasting blood sugar, triglycerides, cholesterol, low-density lipoprotein-cholesterol, and triglycerides/high-density lipoprotein-cholesterol were significantly higher in overweight and obese adolescent groups (*p* <0.0001) in comparison with the normal group. Based on receiver operating characteristics calculation for body mass index and some metabolic risks, the data suggest the best body mass index cut-offs ranged between 23.25 and 24.35 kg/m^2^.

**Conclusions:**

A strong association between overweight and obesity as determined by body mass index and high concentrations of metabolic syndrome components has been demonstrated. Although body mass index values were lower than the international cut-offs, these values were good predictors of some metabolic abnormalities in Syrian adolescents; body mass index is a good predictor of these abnormalities in this population.

## Background

According to the estimates of the World Health Organization (WHO), obesity has become a serious epidemic health problem in many parts of the world, estimated to be the fifth leading cause of mortality at a global level, causing approximately 2.8 million deaths per year [1]. As a result, it was estimated that of the projected 64 million deaths worldwide in 2015, 41 million (64%) will result from chronic disease unless urgent action is taken [2]. Obesity is one of the main components of the metabolic syndrome (MetS), which is a cluster of risk factors for cardiovascular disease (CVD) [3]. These factors not only lead to reduced quality of life given their protracted nature, they also lead to premature death [4].

The risk of diseases related to this syndrome appears to increase as a function of the body fat percentage. For the purpose of risk stratification, it is convenient to have cut-off values of body fat content based on its observed association with disease [5, 6]. A fat mass index was used in screening for the presence of MetS [3]. The American Health Association and other professional societies recommend assessment of obesity using indices such as body mass index (BMI) [7, 8].

Studies have indicated an increase in mortality of various causes with increased BMI, especially death from CVD in men [9]. Increased BMI was most prominently associated with body pain [10]. Overweight and obese young people are viewed by many public health and health care practitioners as needing attention because of the continued focus on biomedical constructions of appropriate body size and its relationship to “good health.” Acceptance of the views that “thin is good” and “fat is bad” is considered paramount for successful public health control [11].

Taking all these issues into consideration, very limited information exists on the prevalence of overweight, obesity, and associated metabolic risk factors among Syrian adolescents. Therefore, the purpose of this study was to determine the relationship between obesity as determined by BMI and the major metabolic risk factors among Syrian adolescents.

## Methods

### Participants

A cross-sectional study consisting of a sample of 2064 healthy young men aged 18 to 19 years from Damascus city, in Syria, was performed. All participants underwent a clinical examination by physicians to exclude those with a clinical history of chronic diseases including cardiovascular, renal, and hepatic or any abnormalities that might affect body composition. Participants were asked to abstain completely from consuming food and drink in the 12 hours before visiting the testing field. All anthropometry measurements and sampling were completed during a single visit to the testing area. The study protocol was approved by the scientific research and the ethical committee of the Atomic Energy Commission of Syria (AECS). Each participant provided informed consent prior to participation after a detailed explanation of the study protocol. This study was performed in accordance with guidelines prescribed by the Declaration of Helsinki as developed by the World Medical Association.

### Anthropometry

Body weight was measured using calibrated electronic scales (Seca, Model 7671321004; Germany; D = 0.05 to 0.1 kg). The accuracy of the scales was confirmed by using weight of known mass (20 kg). Height was measured to the nearest 0.5 cm with a wall-mounted stadiometer (Seca, Model 225 1721009; Germany). Participants were measured in light underwear. BMI was identified as weight divided by height squared (kg/m^2^).

### Biochemical and clinical tests

The main MetS risk factors and some clinically important parameters were included in this study. Systolic blood pressure (SBP) and diastolic blood pressure (DBP) were measured in adolescents in a sitting position after rest using a mercury sphygmomanometer. Blood samples were collected from all participating adolescents after 12 hours of overnight fasting. Serum was separated by centrifugation. Serum glucose (GOD-PAP method, Human Co.), cholesterol (Chol), low-density lipoprotein Chol (LDL-Chol), high-density lipoprotein Chol (HDL-Chol; CHOD-PAP method, Human Co.), triglycerides (TG; GPO-PAP method, Human Co.), and fasting blood sugar (FBS) were determined using commercial kits [12].

### Biochemical, clinical, and overweight/obesity cut-off values

The normal range for each studied metabolic component was defined as follows: SBP (90 to 135 mmHg); DBP (60 to 89 mmHg); FBS (65 to 110 mg/dl); TG (25 to 200 mg/dl); Chol (50 to 200 mg/dl); HDL-Chol (40 to 75 mg/dl); LDL-CDL-Chol (less than 155 mg/dl) [12]. Overweight and obesity were defined as a participant with BMI of 25 to 29.9 kg/m^2^ and ≥30 kg/m^2^, respectively according to the cut-off point recommended by WHO [13]. The normal value for TG/HDL-Chol is less than 3 [14].

### Statistical analysis

Statistical analyses were performed using the Statistical Package for the Social Sciences (SPSS) for windows (Version 17.0.1, 2001, SPSS Inc., Chicago, USA). Continuous variables were expressed as mean ± standard deviation (SD), whereas categorical variables were represented by frequency and percentage. A receiver operating characteristics (ROC) curve was drawn to determine appropriate cut-off points of the BMI for defining overweight and obesity. The area under curve (AUC) with 95% confidence interval (CI) values provided an indication of the performance of BMI as a predictor of health risk [8, 15].

## Results

The mean values (mean ± SD, maximum and minimum) of the MetS components of the 2064 participants and the range of the BMI are illustrated in Table 1. As shown in Table 1, mean serum concentrations of glucose, TG, total Chol, LDL-Chol, TG/HDL-Chol and HDL-Chol were within the normal limits. These values were as follows: BMI (23.14 kg/m^2^), SBP (123.43 mmHg), DBP (74.40 mmHg), FBS (89.49 mg/100 ml), TG (91.84 mg/100 ml), total Chol (136.36 mg/100 ml), HDL-Chol (57.11 mg/100 ml), LDL-Chol (62.04 mg/100 ml), and TG/HDL-Chol (1.61).

**Table 1:** Baseline characteristics of body mass index and biochemical variables in Syrian adolescents (18 to 19 years)

Characteristics	*n*	(Mean ± SD)	Minimum	Maximum
Body mass index (kg/m^2^)	2064	23.14 ± 3.94	16.10	42.90
Systolic blood pressure (mmHg)	2064	123.43 ± 14.55	90.00	180.00
Diastolic blood pressure (mmHg)	2064	74.40 ± 10.72	45.00	105.00
Fasting blood sugar (mg/100 ml)	2064	89.49 ± 8.53	65.00	131.00
Triglycerides (mg/100 ml)	2064	91.84 ± 44.06	12.90	370.00
Total cholesterol (mg/100 ml)	2064	136.36 ± 33.04	17.00	288.00
High-density lipoprotein cholesterol (mg/100 ml)	2064	57.11 ± 5.63	37.00	90.00
Low-density lipoprotein cholesterol (mg/100 ml)	2064	62.04 ± 29.34	1.00	195.00
Triglyceride to high-density lipoprotein cholesterol	2064	1.61 ± 0.78	0.18	6.96
Hematocrit (%)	2064	14.63 ± 0.92	31.00	56.00
Hemoglobin (mg/100 ml)	2064	16.77 ± 6.28	10.00	17.90

Of the 2064 participants, the following had values within the normal range: 1670 (81%) had SBP (90 to 135 mmHg), 1440 (70%) had DBP (60 to 89 mmHg), 2039 (99%) had FBS (65 to 110 mg/dl), 1981 (96%) had Chol (50 to 200 mg/dl), 1989 (96%) had TG (25 to 200 mg/dl), 2034 (99%) had HDL-Chol (40 to 75 mg/dl), 2064 (100%) had LDL-Chol (less than 155 mg/dl), 1950 (95%) had TG/HDL-Chol (less than 3). These results are shown in Table 2.

**Table 2:** Classification of blood pressure and metabolic characteristics in Syrian adolescents (18 to 19 years)

Characteristics		Range	*n*	%
SBP (mmHg)	Lower	<90 mmHg	13	0.6
	Normal	90–135 mmHg	1670	80.9
	Upper	>135 mmHg	381	18.5
DBP (mmHg)	Lower	<60 mmHg	362	17.5
	Normal	60–89 mmHg	1440	69.8
	Upper	>89 mmHg	262	12.7
FBS (mg/100 ml))	Lower	<65 mg/100 ml	1	0.0
	Normal	65–110 mg/100 ml	2039	98.8
	Upper	>110 mg/100 ml	24	1.2
Chol (mg/100 ml)	Lower	<50 mg/100 ml	8	0.4
	Normal	50–200 mg/100 ml	1981	96.0
	Upper	>200 mg/100 ml	75	3.6
TG (mg/100 ml)	Lower	<25 mg/100 ml	17	0.8
	Normal	25–200 mg/100 ml	1989	96.4
	Upper	>200 mg/100 ml	58	2.8
HDL-Chol (mg/100 ml)	Lower	<40 mg/100 ml	20	1.0
	Normal	40–75 mg/100 ml	2034	98.5
	Upper	>75 mg/100 ml	10	0.5
LDL-Chol (mg/100 ml)	Lower	<155 mg/100 ml	2064	100
	Upper	>155 mg/100 ml	0	0.0
TG/HDL-Chol	Lower	<3 mg/100 ml	1950	94.5
	Upper	>3 mg/100 ml	114	5.5

*SBP* systolic blood pressure, *DBP* diastolic blood pressure, *FBS* fasting blood sugar, *TG* triglycerides, *Chol* cholesterol, *HDL-Chol* high-density lipoprotein cholesterol, *LDL-Chol* low-density lipoprotein cholesterol, *TG/HDL-Chol* triglycerides/ high-density lipoprotein cholesterol

Blood pressure and biomedical markers values increased significantly (*p* < 0.05) with increasing BMI value. Regarding the measurements of BMI, the obese group (BMI >30 kg/m^2^) had higher values of SBP (127.3 mmHg), DBP (77.7 mmHg), FBS (91.6 mg/100 ml), TG (125.1 mg/100 ml), Chol (153.6 mg/100 ml), LDL-Chol (71.9 mg/100 ml), and TG/HDL-Chol (2.22) compared to participants who were not obese (20 < BMI <25 kg/m^2^). However, no significant differences were observed for HDL-Chol between the analyzed groups (Table 3).

**Table 3:** Metabolic risk factors in the Syrian adolescents by body mass index category

Characteristics	Underweight BMI <20	Normal 20 < BMI <25	Overweight 25 < BMI <30	Obesity BMI >30	LSD 5%
SBP (mmHg)	121.85 ± 16.60^a^	122.9 ± 14.65^a^	124.2 ± 13.2^a^	127.3 ± 14.55^b^	14.55
DBP (mmHg)	72.8 ± 11.2^a^	73.7 ± 10.6^a^	76.4 ± 10.2^bc^	77.7 ± 11.5^c^	10.72
FBS (mg/100 ml)	90.5 ± 10.5^ab^	89.1 ± 8.4^a^	89.1 ± 8.4^ab^	91.6 ± 7.9^b^	8.53
TG (mg/100 ml)	89.5 ± 51.5^a^	86.9 ± 39.4^a^	99.8 ± 44.6^b^	125.1 ± 62.9^c^	44.06
Chol (mg/100 ml)	132.7 ± 29.6^a^	134.4 ± 32.1^a^	139.0 ± 35.2^b^	153.6 ± 34.6^c^	33.04
HDL-Chol (mg/100 ml)	57.4 ± 5.4^ab^	57.0 ± 5.7^a^	57.7 ± 5.6^b^	56.8 ± 5. 5^ab^	5.63
LDL-Chol (mg/100 ml)	58.9 ± 27.2^a^	61.3 ± 28.8^a^	62.2 ± 30.9^a^	71.9 ± 30.7^b^	29.34
TG/HDL-Chol	1.56 ± 0.91^a^	1.53 ± 0.70^a^	1.74 ± 0.78^b^	2.22 ± 1.15^c^	0.78

*BMI* body mass index, *LSD* least significant difference, *SBP* systolic blood pressure, *DBP* diastolic blood pressure, *FBS* fasting blood sugar, *TG* triglycerides, *Chol* cholesterol, *HDL-Chol* high-density lipoprotein cholesterol, *LDL-Chol* low-density lipoprotein cholesterol, *TG/HDL-Chol* triglycerides/ high-density lipoprotein cholesterol

^a, b, c^mean values in the same row not sharing a superscript are significantly different

ROC curve analysis of the BMI was performed for blood pressure (SBP and DBP) and other lipid biomarkers like FBS, TG, Chol, HDL-Chol, LDL-Chol, and TG/HDL-Chol (Table 4). Area under the ROC curve was the highest for LDL-Chol (0.69%). The BMI cut-off point for LDL-Chol for optimal sensitivity and specificity was (24.35 kg/m^2^) with sensitivity at 60% and specificity at 72%. Comparable ROC curves of other biomarkers were obtained: DBP (AUC 0.59 of optimal cut-off point 23.45 kg/m^2^), TG (AUC 0.67 of optimal cut-off point 23.25 kg/m^2^), Chol (AUC 0.67 of optimal cut-off point 23.75 kg/m^2^), and TG/HDL-Chol (AUC 0.66 of optimal cut-off point 23.45 kg/m^2^). AUC ranged between 0.44 and 0.81 and was statically significant for DBP, TG, Chol, LDL-Chol, and TG/HDL-Chol (Table 4).

**Table 4:** Sensitivity, specificity and area under curve of cut-off value of body mass index indicator in prediction of MetS for Syrian adolescents (18 to 19 years)

Characteristics	Criteria	BMI cut-off value	Sensitivity %	Specificity %	Area under the curve	*P* value
SBP (mmHg)	>135 mmHg	22.85	44.9	58.6	0.52	0.246
DBP (mmHg)	>89 mmHg	23.45	49.6	66.5	0.59	0.000
FBS (mg/100 ml)	>89 mg/100 ml	23.55	37.5	65.2	0.44	0.297
TG (mg/100 ml)	>200 mg/100 ml	23.25	67.2	62.8	0.67	0.000
Chol (mg/100 ml)	>200 mg/100 ml	23.75	61.3	68.2	0.67	0.000
HDL-Chol (mg/100 ml)	<75 mg/100 ml	26.05	30.0	81.3	0.38	0.200
LDL-Chol (mg/100 ml)	<155 mg/100 ml	24.35	60.0	71.9	0.69	0.037
TG/HDL-Chol	>3 mg/100 ml	23.45	62.3	66.0	0.66	0.000

*BMI* body mass index, *SBP* systolic blood pressure, *DBP* diastolic blood pressure, *FBS* fasting blood sugar, *TG* triglycerides, *Chol* cholesterol, *HDL-Chol* high-density lipoprotein cholesterol, *LDL-Chol* low-density lipoprotein cholesterol, *TG/HDL-Chol* triglycerides/ high-density lipoprotein cholesterol

## Discussion

The prevalence of overweight and obesity is increasing rapidly in young populations worldwide, especially in developing countries. Childhood obesity is a major health issue. According to the International Obesity Task Force (IOTF), at least 500 million school children aged 5 to 17 years are overweight or obese. Many studies have reported an association between childhood obesity and the development of a cluster of cardiometabolic disease risk factors [16–19]. This cluster, which has been termed MetS, is associated with the development of diabetes and CVD in both childhood and adulthood; CVD is the leading cause of morality worldwide. In addition, MetS was associated with arterial stiffness, which was a cardiovascular outcome of MetS [3, 20]. Results reported recently indicated that the percentage of individuals who presented MetS risk factors almost doubled over a 10-year period [21]. Therefore, it is very important to develop an effective screening tool of MetS and its individual components, and analyze their association with overweight and obesity. To the best of our knowledge, this is the first study addressing the association of BMI and the components of MetS among Syrian adolescents aged 18 to 19 years.

In this study, a strong association between obesity as defined by BMI and the risk of higher concentrations of blood pressure, FBS, total Chol, TG, HDL-Chol, and TG/HDL in Syrian adolescents has been demonstrated. These findings demonstrate the risk of obesity at these early ages, with changes in the MetS components. Among the metabolic components, mean SBP (123.4 ± 14.55) was elevated in 19% of the whole group. Also, mean DBP (74.4 ± 10.72) was elevated in 12.7%. Increased FBS, TG, Chol, and TG/HDL were present in 1.2%, 2.8%, 3.6%, and 5.5% of those investigated. Their blood pressure, both systolic and diastolic, was the most prevalent of the metabolic risk factors, followed by total Chol and TG. There was a significant increase in blood pressure in the overweight group as compared to the normal group and a significant increase in blood pressure in the obese adolescent group compared to the overweight group (*p* <0.0001). Similar trends were observed for FBS, TG, total Chol, LDL-Chol, and TG/HDL-Chol. We also observed that the metabolic abnormalities were more significant and frequent in individuals with higher BMI values, especially in obese adolescents compared to those considered to have normal weight or overweight. Obese adolescents had higher abdominal fat, which is associated with cardiometabolic alterations such as LDL-Chol, increased triglycerides and LDL-Chol, and increased blood pressure, resulting in increased risk for CVD [22].

Several studies have demonstrated that overweight and obesity in childhood are risk factors for coronary disease and MetS in adulthood. In addition, hyperinsulinemia and hypertension are well-known risk factors for coronary heart disease. Our results show that these risk factors are present in a significant number of Syrian adolescents, mainly in those who are obese. Our data showed that overweight and obesity were associated with increased values in the components of MetS. These results are in agreement with the data of other studies investigating a similar association between the components of MetS and obesity [23–27].

Anthropometric measurements are crucial for MetS diagnosis. In addition, measurements of serum lipid fractions, fasting glucose values, and blood pressure in overweight and obese individuals are also important [28].

In this study, BMI was used to screen for the presence of MetS. Other studies concluded that BMI can predict the presence of multiple metabolic risk factors [29]. However, some studies found that a high body fat percentage was associated with increased cardiovascular risk regardless of BMI whose categorization resulted in an underestimation of the number of participants with cardiovascular risk factors [30, 31].

CVD is the most common cause of death in the world. The identification of individuals at increased CVD risk represents a priority [32]. Among the metabolic components, mean SBP (123.4 ± 14.55) was elevated in 19% of the whole group. Also, mean DBP (74.4 ± 10.72) was elevated in 12.7%. Increased FBS, TG, Chol, and TG/HDL-Chol were present in 1.2%, 2.8%, 3.6%, and 5.5% of those investigated. Data from the present study suggested that populations who have high BMI, high SBP and DBP, and high levels of serum FBS, TG, Chol, LDL-Chol, and TG/HDL-Chol are more likely to experience a cardiovascular effect. In fact, LDL-Chol, which is closely related to CVD and mortality, remains the cornerstone of lipid management [33]. Epidemiological and clinical studies have consistently demonstrated that increased LDL-Chol is a major atherogenic lipoprotein for developing CVD and is currently recommended as the primary target for lipid-lowering therapy for the prevention and treatment of CVD [32, 34]. In some other studies, a significant relationship was found between cardiorespiratory fitness, BMI, and LDL-Chol [35]. Epidemiological investigations revealed that the occurrence of CVD in the obese population was 2.0 to 2.5 times that in people with a normal BMI [36]. The risk of hypertension is up to five times higher among obese people in comparison to normal weight individuals, and cross-sectional population surveys suggest that more than 85% of hypertension arises in individuals with BMI values above 25 kg/m^2^ [24]. Our findings were in line with reports from many previous studies; Chen *et al*. [25], Mokdad *et al*. [26], and Wilson *et al*. [27] demonstrated that an increased risk of obesity-related morbidities was accompanied by an increase in BMI. Among the indicators used to predict the presence of MetS, TG, Chol, LDL-Chol, and TG/HDL-Chol showed a large area under the ROC curve, but LDL-Chol was the index that showed the greatest area under the ROC curve, which was similar to other studies [3, 32]. Based on the sensitivity, specificity, and ROC calculation, we found that BMI had a good accuracy for identifying adolescents with some metabolic risk factors including DBP, TG, Chol, LDL-Chol, and TG/HDL-Chol; these data suggest the best BMI cut-offs ranged between 23.25 and 24.35 kg/m^2^. Our cut-off values of BMI were lower than the current definitions of overweight (BMI >25) recommended by WHO [1, 13]. These figures are in agreement with data from Asian populations [6], but lower than Western populations in USA and Europe [5]. On the other hand, our findings differ from those found in the Iraqi population (in men) [37]; they found that the best BMI cut-offs ranged between 24.9 and 25.4.

However, many studies have shown that CVDs occur more frequently at lower BMI in Asian than European populations, the mechanisms involved are yet to be demonstrated. Ethnic and racial differences in body composition and fat distribution have been studied. Although these studies have shown increasing BMI associated with larger increase in body fat content in Asians, growing evidence points to factors other than body fat content and fat distribution in determining higher prevalence of CVDs in these populations [38]. In a study on young adult Mauritians, the authors pointed out that the use of BMI cut-off values for classifying overweight and obesity should take into account both ethnicity and gender to avoid gross adiposity status misclassification in this population known to be at high risk for CVDs [39].

This study, however, brings an important epidemiological contribution to Syria, revealing important results, especially due to the lack of studies that evaluate the frequency of cardiovascular risk factors among Syrian adolescents and the value of BMI in predicting the components of MetS. Our findings support the notion of a relevant association between obesity at these early ages and the development of alterations in the components of MetS. Therefore, the planning and implementation of primary prevention programs by health care professionals to reduce potential cardiovascular risk factors is a priority. By such programs CVD risk factors can be reduced. Even small reductions in body weight are able to correct abnormalities in the components of MetS, such as hyperglycemia, hyperinsulinemia, and dyslipidemias. Interventions aimed at increasing physical activity and improving diet are cost effective for reducing the risk of diabetes and CVDs associated with obesity.

## Conclusions

In conclusion, a strong association between overweight and obesity as determined by BMI and high concentrations of MetS components has been demonstrated in overweight and obese Syrian adolescents. BMI categories of >23.45 kg/m^2^, >23.25 kg/m^2^, >23.75 kg/m^2^, >24.35 kg/m^2^, and >23.45 kg/m^2^ were found to be positively associated with MetS components of DBP, TG, Chol, LDL-Chol, and TG/HDL-Chol, respectively. These findings demonstrate the high risk of obesity at an early age through abnormal changes in MetS components.
